# A Case of Traumatic Cerebrospinal Fluid Rhinorrhea Successfully Treated Using Intravenous Factor XIII Administration

**DOI:** 10.7759/cureus.15633

**Published:** 2021-06-14

**Authors:** Iori Yasuda, Masahito Katsuki, Norio Narita

**Affiliations:** 1 Department of Neurosurgery, Kesennuma City Hospital, Kesennuma, JPN; 2 Department of Neurosurgery, Itoigawa General Hospital, Itoigawa, JPN

**Keywords:** blood coagulation factor xiii, cerebrospinal fluid leakage, neurotrauma, lumbar spinal drainage, traumatic cerebrospinal fluid rhinorrhea

## Abstract

Traumatic cerebrospinal fluid (CSF) rhinorrhea occurs around 2% of severe head trauma. We should find the fistula and surgically seal it or perform conservative therapy with bed rest with/without lumbar spinal CSF drainage. However, the fistula may not be identified, and treatment may sometimes be challenging. Blood coagulation factor XIII (factor XIII) is one of the blood coagulation factors. It also promotes fibroblast proliferation during the wound healing process. We herein reported a traumatic CSF rhinorrhea patient who was successfully treated using intravenous (IV) factor XIII administration. This report would contribute to the effectiveness of factor XIII administration in the treatment of traumatic CSF rhinorrhea. A 58-year-old man fell from a height of 1.5 meters and hit his forehead. He presented with numbness in both upper limbs but no paresis. Neck magnetic resonance imaging (MRI) revealed cervical spinal cord injury without a cervical vertebral or cranial fracture. He was conservatively treated and discharged after three months. He had been aware of rhinorrhea since the trauma but was treated as allergic rhinitis. A year after the trauma, he was diagnosed with traumatic CSF rhinorrhea. We confirmed a bit of rhinorrhea despite the seven-day bedrest, so we intravenously administered 240 international units of factor XIII every day for 10 days. After 10 days, there was no rhinorrhea at all, and the patient was discharged on the 28th day. He has had no recurrence of rhinorrhea after a three-month follow-up. Factor XIII administration might be useful to treat traumatic CSF rhinorrhea.

## Introduction

Traumatic cerebrospinal fluid (CSF) rhinorrhea occurs in around 2% of severe head trauma [1]. CSF rhinorrhea means CSF communication between the nasal sinuses and the intracranium, which may cause intracranial infections, meningoencephalocele, or pneumocephalus [2]. Therefore, we should find the fistula and surgically seal it [3] or perform conservative therapy with bed rest with/without spinal CSF drainage [4-5]. However, the fistula may not be identified, and treatment may sometimes be challenging.

Blood coagulation factor XIII (factor XIII) is one of the blood coagulation factors found by Laki [6]. It not only catalyzes the formation of cross-links between fibrin molecules during the final stages of coagulation but also promotes fibroblast proliferation during the wound healing process [7]. Its efficacy in treating post-neurosurgical CSF leakage has been reported, but the reports remain few [8-9]. Furthermore, a case with “traumatic” CSF rhinorrhea treated by factor XIII has not been reported.

We herein report a traumatic CSF rhinorrhea patient who was successfully treated using intravenous factor XIII administration. This report would contribute to the effectiveness of factor XIII administration in the treatment of traumatic CSF rhinorrhea.

## Case presentation

**Figure 1:**
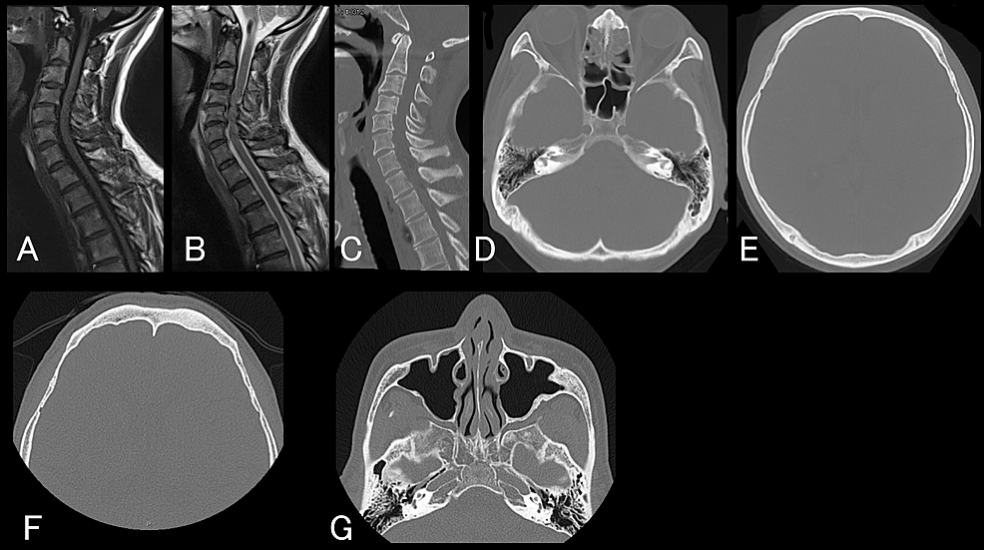
Images at the time of trauma and one year from the trauma Upper row (images at the time of trauma): T1-weighted (A) and T2-weighted (B) magnetic resonance imaging. They revealed cervical spinal cord injury without cervical vertebral fracture (C). Head computed tomography (CT) images did not suggest cranial fracture nor pneumocephalus (D, E). Lower row: CT images still did not reveal a fracture, fistula, pneumocephalus, nor injury of the dura (F, G).

A 58-year-old man fell from a height of 1.5 meters and hit his forehead during fishing work. He presented with numbness in both upper limbs but no pareses. Neck magnetic resonance imaging (MRI) and neck and head computed tomography (CT) revealed cervical spinal cord injury without cervical vertebral or cranial fractures (Figures 1A-1E). He was admitted to the orthopedics department, treated conservatively, and discharged home after a three-month rehabilitation. He had been aware of rhinorrhea since the trauma but was treated as allergic rhinitis. Notably, he was first treated on bed rest for two weeks due to cervical cord injury, but the rhinorrhea did not improve despite the bedrest term. A year after the trauma, he consulted an otolaryngologist, and the otolaryngologist, for the first time, referred him to us to distinguish CSF rhinorrhea. The nasal endoscopic findings by the otolaryngologist could not find an apparent fistula. The urine test paper revealed that the glucose level in the nasal discharge was about 50-100 mg/dL, suggesting CSF rhinorrhea [2]. He did not have a fever, headache, or stiff neck. The laboratory test results of blood were all within normal limits. CT and MRI did not elucidate fistula, fractures, pneumocephalus, or injury of the dura (Figures 1F-1G).

He was admitted to the neurosurgical ward, and we had him keeping bedrest. His factor XIII activity level was decreased by 72%. We confirmed a bit of rhinorrhea during bed rest despite the seven-day bedrest. We then intravenously administered 240 international units of factor XIII every day for 10 days. The rhinorrhea stopped on the third day. After 10 days of the administration, he gradually started to sit, stand, and walk for four days. There was no rhinorrhea at all, and the patient was discharged on the 28^th^ day. He has had no recurrence of rhinorrhea after a five-month follow-up.

## Discussion

We report a case with traumatic CSF rhinorrhea successfully treated with intravenous factor XIII administration. This is the first report on the utility of factor XIII for traumatic CSF rhinorrhea with undetermined fistula.

Conservative treatment for traumatic CSF rhinorrhea

Recently, CSF rhinorrhea tends to be treated conservatively [10]. There are two opinions about the position: head-up and head-down. Head-up may cause less ascending infection and contribute to sealing the fistula with the brain’s weight to promote healing. On the other hand, in head-down, the bridging veins are not pulled by the brain due to gravity. Also, head-down may reduce the pressure gradient between the intracranial and intraspinal portions and prevent tension pneumocephalus. If spinal drainage is performed [4], it may be possible to promote healing by eliminating CSF communication via the fistula. However, drainage may adversely increase the risk of ascending infection or cause tension pneumocephalus [5].

In such a yet-to-be-established treatment method for traumatic CSF rhinorrhea, factor XIII administration could be another conservative treatment for CSF rhinorrhea, although infections and allergies could occur as side effects.

Factor XIII

Factor XIII is known as the fibrin-stabilizing factor. It is an enzyme that proliferates fibroblasts and plays a major role in granulation during the wound healing process [7]. It is known that surgery and hemorrhage can decrease factor XIII activity level and delay wound healing. In neurosurgery, Gerlach reported that factor XIII activity levels were low in patients with postoperative hemorrhage after surgery with craniotomy [11]. Kawamura reported CSF leakage after the removal of a large meningioma at the cerebellopontine angle using the transpetrosal approach. The patients were first treated by spinal drainage for two weeks, but the leakage did not stop. The factor XIII activity level was less than 30%, so they administered factor XIII. The CSF leakage was cured seven days after administration [8]. Nakano reported CSF leakage after clipping for a ruptured aneurysm at the anterior communicating artery by the basal interhemispheric approach. The patients were treated by spinal drainage and factor XIII administration simultaneously. The factor XIII activity level was 68%, and the CSF leakage was cured three days after administration [9].

As described above, the utility of factor XIII has been reported to cure the post-neurosurgical CSF leakage. Also, factor XIII seemed useful for spontaneous intracranial hypotension treatment [7]. In addition to these reports, our case suggests that factor XIII administration may be effective for traumatic CSF leaks without apparent fistula, even the XIII activity level is not less than 70%.

Limitation

We did not test the beta-2-transferrin level in the nasal discharge and perform radionuclide cisternography specific for CSF [2]. Also, it is unclear whether CSF rhinorrhea was cured by factor XIII administration for 10 days or bed rest for 17 days. Further research is warranted to establish factor XIII administration as a treatment method for traumatic CSF rhinorrhea. Finally, factor XIII administration is not covered by the Japanese national insurance system without a decrease in factor XIII activity level by 70%. It is used for bleeding tendency due to congenital and acquired factor XIII deficiency, suture failure, and fistula due to factor XIII deficiency and immunoglobulin A (IgA) vasculitis. The side effects and contraindications are allergies for factor XIII administration.

## Conclusions

We herein reported a 58-year-old man with traumatic CSF rhinorrhea who was successfully treated using intravenous factor XIII administration for 10 days. It is unclear whether CSF rhinorrhea was cured by factor XIII administration or bed rest for 17 days. Further research is warranted to establish factor XIII administration as a treatment method for traumatic CSF rhinorrhea. Also, the side effect and contraindications are other problems for factor XIII administration. However, this report would contribute to the effectiveness of factor XIII administration as an alternative treatment of traumatic CSF rhinorrhea.
